# The trafficking and targeting of P2X receptors

**DOI:** 10.3389/fncel.2013.00233

**Published:** 2013-11-22

**Authors:** Lucy E. Robinson, Ruth D. Murrell-Lagnado

**Affiliations:** Department of Pharmacology, University of CambridgeCambridge, UK

**Keywords:** P2X receptor trafficking, lipid rafts, receptor endocytosis, receptor regulation, P2X receptor targeting

## Abstract

The functional expression of P2X receptors at the plasma membrane is dependent on their trafficking along secretory and endocytic pathways. There are seven P2X receptor subunits, and these differ in their subcellular distributions because they have very different trafficking properties. Some are retained within the endoplasmic reticulum (ER), while others are predominantly at the cell surface or within endosomes and lysosomes. Changes in recruitment of receptors to and from the plasma membrane provides a way of rapidly up- or down-regulating the cellular response to adenosine triphosphate (ATP). An additional layer of regulation is the targeting of these receptors within the membranes of each compartment, which affects their stability, function and the nature of the effector proteins with which they form signaling complexes. The trafficking and targeting of P2X receptors is regulated by their interactions with other proteins and with lipids and we can expect this to vary in a cell-type specific manner and in response to changes in the environment giving rise to differences in receptor activity and function.

## Introduction

P2X receptors open an integral ion channel at the plasma membrane in response to binding extracellular adenosine triphosphate (ATP). Some subtypes of P2X receptor are predominantly within intracellular membranes, but there is no compelling evidence that the receptors on intracellular membranes open in response to ATP, at least in mammals. Nonetheless, this remains an intriguing possibility, given that the distantly related P2X-like receptors in *Dictyostelium* are located and function within contractile vacuoles (Fountain et al., 2007; Ludlow et al., 2009; Sivaramakrishnan and Fountain, 2012a,b; Baines et al., 2013). The best-established role of the internal mammalian P2X receptors is, therefore, to regulate the expression and activity of receptors at the cell surface. Here we consider three related issues concerning the targeting and trafficking of P2X receptors: first, primary location, and the amino acid motifs which determine it; second, regulation of mobility both within the plasma membrane and between the plasma membrane and intracellular membranes; third, targeting to lipid rafts and the effects of the lipid environment on receptor signaling.

## Subcellular localization of P2X receptors

Trimeric P2X receptor complexes assemble and are core glycosylated within the endoplasmic reticulum (ER) and then traffic via the trans-Golgi network (TGN) to the plasma membrane.

They are subsequently internalized and either recycled back to the surface or targeted to late endosomes and lysosomes. The kinetics of these processes determines receptor distribution.

### ER resident P2X receptors

**Figure 1:**
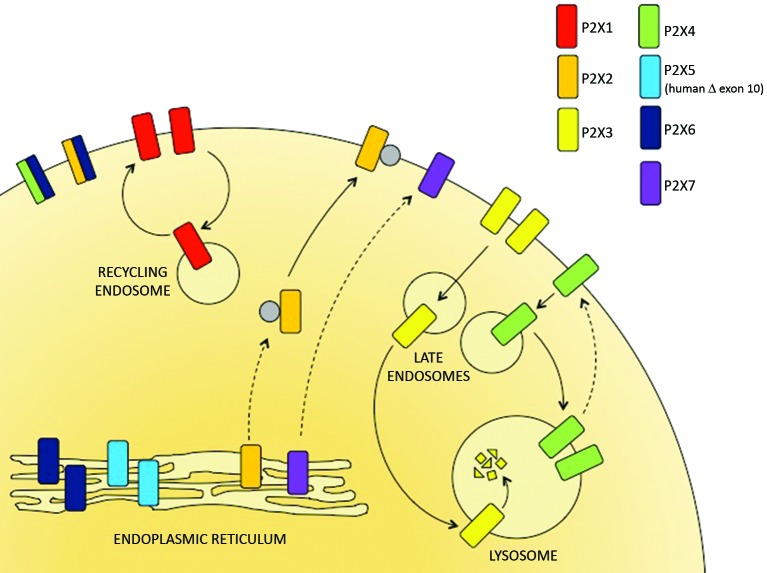
**The subcellular distribution of P2X receptors.** P2X receptor subtypes differ in their trafficking properties and hence are localized to different subcellular compartments. P2X6 receptors are retained within the ER but can assemble with P2X4 and P2X6 subunits to form heterotrimers that traffic to the cell surface. The predominant human allele of P2X5 lacks exon 10 and is also retained in the ER. P2X2 and P2X7 receptors traffic relatively slowly through the secretory pathway but are stably expressed at the surface. P2X1 receptors are expressed at the cell surface but rapidly cycle to and from recycling endosomes. P2X3 and P2X4 receptors are consitutively internalized and delivered to late endosomes and lysosomes. Within the lysosomes, P2X3 receptors are rapidly degraded but P2X4 receptors resist degradation and can recycle back to the surface.

P2X receptors are predominantly found within the ER, at the plasma membrane or within late endosomes and lysosomes, dependent upon the subtype (Figure 1). The only full-length P2X receptor that is retained within the ER and is therefore non-functional is P2X6 (Ormond et al., 2006). Imaging of P2X6 receptors by atomic force microscopy indicates that the subunits do not assemble to form stable homotrimeric complexes, but they do form stable heterotrimers with either P2X2 or P2X4 (Bobanovic et al., 2002; Barrera et al., 2005, 2007; Ormond et al., 2006). The P2X2/6 and P2X4/6 receptors are expressed as functional receptors at the plasma membrane and have trafficking properties that resemble the P2X2 and P2X4 homomeric receptors respectively. In the category of ER resident P2X receptors there is also the human P2X5 receptor. Although the full-length P2X5 receptor traffics to the cell surface, the predominant allele expressed in most humans gives rise to an exon 10-deleted variant which is retained in the ER (Bo et al., 2003; Kotnis et al., 2010; Compan et al., 2012).

### Plasma membrane P2X receptors

Two subtypes that traffic relatively slowly through the secretory pathway and hence often appear to have a predominantly ER distribution are P2X2 and P2X7 receptors. P2X2 receptors are stably expressed at the plasma membrane, but, when heterologously expressed, they accumulate slowly at the cell surface. This slow traffic might be important for facilitating interactions with other proteins along the way (Bobanovic et al., 2002). For example, in spinal cord neurons, intracellular P2X2 receptors interact with GABA_A_ receptors and co-traffic to the surface (Shrivastava et al., 2011). Another protein that interacts with P2X2 receptors to regulate its targeting to synapses is the beta-amyloid precursor protein-binding protein Fe65 (Masin et al., 2006). There is also the neuronal calcium sensor, visinin-like protein-1 (VILIP-1) that interacts with P2X2 in a calcium-dependent manner (Chaumont et al., 2008). These interacting proteins affect the stability, targeting and function of the receptors at the plasma membrane.

P2X7 receptor trafficking is dependent upon cell-type and species. For example, in human monocytes and lymphocytes, P2X7 receptors are predominantly intracellular, but upon differentiation of monocytes to macrophages receptors locate to the plasma membrane (Hickman et al., 1994; Gu et al., 2000; Gudipaty et al., 2001). Native P2X7 receptors in rodent microglia and macrophages are also predominantly at the plasma membrane (Boumechache et al., 2009). What regulates the rate at which P2X7 receptors traffic from the ER to the cell surface is unknown, although mutagenesis analysis suggests that it involves the cytoplasmic C-terminal domain of the receptor (Denlinger et al., 2003; Smart et al., 2003).

P2X1 receptors are predominantly at the cell surface, and at least one interacting protein has been identified, namely heat shock protein 90, which further promotes their trafficking and plasma membrane expression (Lalo et al., 2010, 2012). At the plasma membrane, P2X1 receptors differ in trafficking behavior from P2X2 receptors. P2X2 receptors are relatively stable and show little constitutive internalization over a period of an hour (Bobanovic et al., 2002). In contrast, measurements of P2X1 receptor mobility by fluorescence recovery after photobleaching (FRAP) shows that receptors undergo considerable internalization and recycling over a similar time period (Lalo et al., 2010). Their surface localization indicates that recycling is rapid compared to their rate of endocytosis and targeting to late endosomes.

### Endo-lysosomal P2X receptors

P2X3 and P2X4 receptors also undergo rapid constitutive internalization from the plasma membrane, but unlike P2X1 they are predominantly localized to late endosomes and lysosomes (Bobanovic et al., 2002; Qureshi et al., 2007; Vacca et al., 2009). For P2X3 receptors this has been shown for heterologously expressed receptors in HEK293 cells and also for native receptors in dorsal root ganglion (DRG) neurons (Vacca et al., 2009). For P2X4 receptors, their localization to endolysosomes has been shown in immune and endothelial cells as well as for the heterologously expressed receptors in neurons (Bobanovic et al., 2002; Royle et al., 2002; Qureshi et al., 2007; Toulme et al., 2010). Constitutive endocytosis of P2X4 receptors is mediated via a dynamin/clathrin-dependent process and can be inhibited using dynasore (Bobanovic et al., 2002). Treatment with dynasore rapidly up-regulates P2X4 receptors at the surface of some but not all cells that express this receptor (Boumechache et al., 2009). In cultured microglial cells, native P2X4 receptors are rapidly up-regulated, indicating that they are continually cycling to and from the surface. In contrast, in bone marrow derived macrophages, total P2X4 receptor expression is high, but expression at the surface is very low and not increased by 1 h incubation with dynasore, suggesting that under basal conditions, receptors remain within endolysosomal compartments.

P2X3 receptors have a high rate of turnover, caused by rapid endocytosis and targeting to lysosomes, where the receptor is degraded (Vacca et al., 2009). In contrast, P2X4 receptors are surprisingly stable: they are resistant to degradation in the lysosome and they show little turnover over a period of 24 h (Qureshi et al., 2007). Their resistance to degradation is dependent upon multiple N-linked glycans which decorate the loop between the first and second transmembrane domains (TMD), which is predicted to face towards the lumen of lysosomes. Glycosylation is thought to play a similar role in protecting other lysosome-targeted proteins, such as Lamp-1, from degradation (Kundra and Kornfeld, 1999).

## P2XR motifs that determine receptor trafficking

P2X receptors share a YXXXK in the C-terminus which regulates surface expression (Chaumont et al., 2004). This motif is situated eight residues downstream of TMD2, and in P2X7 after an additional 18 amino acid cysteine-rich region. For P2X2 receptors, mutations around this motif reduce the stability of the receptor at the plasma membrane and increase internalization. Mutations within the motif similarly reduce the plasma membrane expression of other P2X receptor subtypes (Chaumont et al., 2004).

The P2X4 receptor has two tyrosine-based endocytic motifs within the C-terminus and one di-leucine-like motif within the N-terminus (Royle et al., 2002, 2005; Qureshi et al., 2007). Mutations of Y378, which forms part of a non-canonical YXXGΦmotif, substantially slow receptor endocytosis, suggesting that this is the more accessible of the two tyrosine-based motifs (Royle et al., 2002, 2005). These mutants still, however, show targeting to lysosomes, but this is further inhibited by mutating the leucine and isoleucine pair within the N-terminus.

Though the P2X3 receptor does not share any endocytic motifs with P2X4, there is a di-leucine motif in its C-terminal tail and also a consensus sequence for ubiquitination (DSGΦXS) that is suggested to be involved in the endocytosis and rapid degradation of the receptor (Vacca et al., 2009).

For P2X7 receptors, several mutations, deletions and naturally occurring single nucleotide polymorphisms (SNPs) within the long C-terminal tail have been shown to disrupt its normal trafficking to the plasma membrane. Attention has mostly focused on the distal end of the C-terminus of P2X7 (573–590), where there is a region with strong amino acid identity to the lipopolysaccharide (LPS) binding region of the LPS binding protein (Denlinger et al., 2001). Truncations and mutations within a region overlapping this site (551–581) disrupt normal receptor trafficking in HEK293 cells (Smart et al., 2003). For example, the I568N SNP in human P2X7 receptors disrupts normal trafficking and function (Wiley et al., 2003), as does substitution of acidic residues for the dibasic R578 and K579 (Denlinger et al., 2003). More recently it was shown that mutations within this region also disrupt the normal targeting of rat P2X7 receptors in polarized epithelial cells (Bradley et al., 2010). Alanine substitutions at P582-Q587 switched receptor targeting from the basolateral to the apical membrane but without disrupting plasma membrane expression or receptor function. Although site-directed mutagenesis has revealed critical residues in P2X7 receptor trafficking, the mechanism(s) underlying their involvement remains unknown.

## Regulation of receptor trafficking and mobility

The activation of P2X receptors regulates their trafficking to and from the plasma membrane and their mobility within the plasma membrane in a calcium-dependent manner. Agonist-stimulated P2X receptor internalization and recycling back to the plasma membrane was first shown for P2X1 in rat vas deferens (Ennion and Evans, 2001). A later study of P2X1 mobility in HEK293 cells showed an increase in the rate of FRAP at the cell surface in the presence of agonist, which was dependent upon a rise in calcium, upon clathrin-mediated endocytosis and upon trafficking of vesicles back to the surface (Lalo et al., 2010). P2X1 receptors rapidly desensitize in the presence of agonist and inhibiting the internalization and recycling of receptors reduces the rate of recovery from desensitization. The P2X3 receptor also rapidly desensitizes and shows agonist-stimulated internalization (Vacca et al., 2009). P2X4 receptors desensitize more slowly but inhibiting dynamin-mediated endocytosis similarly slows the resensitization process (Murrell-Lagnado, unpublished). Thus, receptor retrieval and recycling appears to be important for maintaining the activity of the surface receptors.

Enhanced translocation of intracellular receptors to the plasma membrane has been shown to be a mechanism for up-regulating receptor function, particularly for those receptors that are predominantly intracellular. P2X4 receptors translocate from endolysosomes back to the surface, whereas for P2X3 receptors it is unclear whether up-regulation involves increased delivery from the secretory or endocytic pathway. ATP produces a transient increase in the number of P2X3 receptors at the surface causing sensitization of the current to repetitive doses (Vacca et al., 2009). Another example of increased trafficking of P2X3 receptors to the plasma membrane is in trigeminal sensory neurons in response to calcitonin gene-related peptide (CGRP; Fabbretti et al., 2006). A 1 h incubation with CGRP increased both the amplitude of P2X3 receptor currents and their rate of recovery from desensitization. For P2X4 receptors in macrophages, surface expression is increased in response to stimuli that promote lysosome exocytosis either by increasing cytosolic calcium or by alkalinization of the lysosomes (Qureshi et al., 2007). In the cerebellar microglial cell line, C8-B4, P2X4 receptor currents are negligible in resting cells, but after activating cells with either LPS or fibronectin, receptors translocate from lysosomes to the surface to enhance receptor-mediated currents (Toulme et al., 2010). Anti-depressants, which inhibit these currents, act by blocking this translocation process rather than by directly inhibiting the opening of the channel pore (Toulme et al., 2010). This mode of action could prove to be a useful way of selectively targeting the different subtypes with new therapeutics.

It is not only the retrieval and recycling of P2X receptors that is sensitive to agonist stimulation: their mobility within the lateral plane of the plasma membrane is promoted by the binding of ATP triggering a local influx of calcium. P2X2 receptor mobility was measured in hippocampal neurons by imaging single molecules using a quantum dot-based approach, and a similar approach was used with P2X4 receptors in microglia (Richler et al., 2011; Toulme and Khakh, 2012). In both cases two populations of receptor were observed, characterized as the mobile and the slowly mobile pool. Neither population correlated with clusters of receptors or receptors localized in lipid rafts, so the molecular basis for the different mobility is unclear. Both populations showed increased mobility in response to ATP. For P2X2 receptors, mobility was also increased by the co-expression of VILIP-1 (Richler et al., 2011). The implications of this increased lateral mobility for receptor signaling remains to be established.

## Targeting of P2X receptors to lipid rafts

The plasma membrane is an extremely heterogeneous environment and the trafficking and function of P2X receptors are regulated by the proteins and lipids within their immediate environment, with which they interact. Lipid rafts are commonly defined as microdomains rich in cholesterol, sphingolipids and saturated phospholipids, but there is heterogeneity amongst these domains in terms of their protein and lipid composition (Pike, 2004). While some proteins are targeted to rafts, others are excluded, affecting the nature of the signaling complexes formed and their stability within the membrane.

Lipid rafts are often identified biochemically by their low buoyant density in a sucrose density gradient, their resistance to solubilization in Triton-X 100 and the presence of protein markers such as caveolin-1 (Pike, 2004). Several of the P2X receptors have been shown to associate with lipid rafts, but the degree to which they distribute between the raft and non-raft fractions depends upon the cells in which they are expressed and the method used to prepare the rafts (Allsopp et al., 2010). P2X1-4 receptors expressed in HEK293 cells associate with rafts prepared using a detergent-free method (Allsopp et al., 2010). When rafts are instead prepared using Triton-X 100, the receptors shift to non-raft fractions. P2X1 and P2X2 receptors are more resistant to extraction from rafts by Triton-X 100 than are P2X3 and P2X4 receptors, suggesting that P2X1 and P2X2 interact more strongly with the cholesterol enriched domains (Allsopp et al., 2010).

Native P2X receptors have also been shown to target to lipid rafts. For P2X1 receptors, the distribution between raft and non-raft fractions is dependent upon the cell type. For example, P2X1 receptors in smooth muscle preparations from artery, vas deferens and bladder are almost exclusively in rafts, whereas only 20% of P2X1 receptors in platelets are in rafts (Vial and Evans, 2005; Vial et al., 2006). Native P2X3 receptors in trigeminal sensory neurons target to lipid rafts and, in a transgenic migraine mouse model, up-regulation of these receptors correlates with an increase in the abundance of lipid rafts and an increase in the fraction of P2X3 receptors within rafts (Gnanasekaran et al., 2011). Thus regulation of lipid rafts provides a mechanism for changing the functional expression of these receptors. P2X7 receptors are also found within rafts, both for heterologously-expressed receptors in HEK293 cells and for native receptors in rat submandibular glands, peritoneal macrophages and mouse lung alveolar cells (Garcia-Marcos et al., 2006a,b; Barth et al., 2007, 2008; Gonnord et al., 2009). Similar to P2X1-4, the association of P2X7 receptors with rafts is dependent upon the method used for isolating them. The receptors target to rafts prepared in detergent-free conditions, but this is reduced by low concentrations of Triton-X-100. This might reflect a weak association of P2X7 receptors with rafts or a difference in the nature of the rafts isolated by these methods. Rafts prepared in the absence of detergent retain a greater fraction of inner leaflet-membrane lipids, in particular phosphatidylserine (Pike et al., 2002) and this might be involved in stabilizing the association with P2X7 receptors.

How P2X receptors target to rafts is unclear. For P2X7 receptors there is evidence to support the involvement of both caveolin-1 and palmitoylation of the receptor within its cytoplasmic C-terminal domain (Barth et al., 2007, 2008; Gonnord et al., 2009). P2X7 receptors palmitoylated with a radiolabeled palmitate were detected exclusively in lipid rafts, while inhibiting palmitoylation reduced receptor targeting to rafts. In mouse lung alveolar epithelial cells, P2X7R and caveolin-1 were detected in the same native complexes and caveolin-1 co-immunoprecipitated with P2X7 receptors (Weinhold et al., 2010). Also, epithelial cells from the caveolin-1 knock-out mice showed reduced levels of P2X7 immunostaining at the plasma membrane (Barth et al., 2007, 2008). A role for caveolin-1 could explain cell-type dependent differences in P2X7 receptor trafficking and function, such as between fibroblasts, rich in caveolin-1, and some immune cells, deficient in caveolin-1.

Depleting plasma membrane cholesterol with methyl-β-cyclodextrin disrupts lipid rafts and alters the function of some but not all of the P2X receptors. P2X1 receptor currents are strongly inhibited by cholesterol depletion, whereas P2X2 receptor currents are unchanged (Vial and Evans, 2005; Allsopp et al., 2010). A region within the N-terminus of P2X1 proximal to TMD1 was identified as an important determinant of cholesterol sensitivity (Allsopp et al., 2010). Cholesterol sensitivity of P2X1 receptor currents was, however, abolished by a cytoskeletal stabilizing agent, suggesting that lipid rafts regulate P2X1 by affecting its interaction with the cytoskeleton (Lalo et al., 2011). P2X3 receptor currents in trigeminal sensory neurons were inhibited by methyl-β-cyclodextrin treatment and desensitization was accelerated (Gnanasekaran et al., 2011). P2X4 receptor currents in Thp-1 monocytes were similarly inhibited by depleting cholesterol (Li and Fountain, 2012). The role of lipid rafts as regulators of P2X7 receptor signaling is of particular interest because receptor stimulation activates lipid-metabolizing enzymes, including phospholipases and sphingomyelinases, that reside in lipid rafts and whose substrates are also enriched in rafts. In cells from rat submandibular glands, cholesterol depletion reduced ATP-stimulated ceramide generation and phospholipase A2 activation, consistent with the idea that targeting to rafts controls signaling between the P2X7 receptor and its downstream effectors (Garcia-Marcos et al., 2006a).

## Summary and outlook

For many of the P2X receptors we now have a basic understanding of their trafficking properties and subcellular distributions, and in some cases have identified regulators that can alter their trafficking to and from the plasma membrane. For the P2X4 receptor, a key unanswered question is why it stably resides within endolysosomes. Only for the type II alveolar cells has a role for P2X4 receptors in the lamellar bodies, which resemble secretory lysosomes, been demonstrated, and only upon their fusion with the plasma membrane (Miklavc et al., 2011). It remains to be established whether P2X4 receptors have additional roles within conventional or secretory lysosomes of other cells. P2X7 receptors are known to be up-regulated in many different cell types under inflammatory conditions, contributing to pathology (Lister et al., 2007). We would like to understand what regulates the trafficking of these receptors to and from the cell surface. For all of the P2X receptors we would like to understand how the lipid environment controls their function and stability. For P2X7 receptors this interaction might also operate in the reverse direction: events downstream of P2X7 receptor activation, including sphingomyelinase activation and ceramide generation directly modify raft structure, thereby providing the potential for cross-talk with other receptors that are modulated by lipid rafts.

## Conflict of interest statement

The authors declare that the research was conducted in the absence of any commercial or financial relationships that could be construed as a potential conflict of interest.
